# Involvement of histone deacetylase CsHDA2 in regulating (*E*)-nerolidol formation in tea (*Camellia sinensis*) exposed to tea green leafhopper infestation

**DOI:** 10.1093/hr/uhac158

**Published:** 2022-07-28

**Authors:** Dachuan Gu, Shuhua Wu, Zhenming Yu, Lanting Zeng, Jiajia Qian, Xiaochen Zhou, Ziyin Yang

**Affiliations:** Guangdong Provincial Key Laboratory of Applied Botany & Key Laboratory of South China Agricultural Plant Molecular Analysis and Genetic Improvement, South China Botanical Garden, Chinese Academy of Sciences, No. 723 Xingke Road, Tianhe District, Guangzhou 510650, China; Guangdong Provincial Key Laboratory of Applied Botany & Key Laboratory of South China Agricultural Plant Molecular Analysis and Genetic Improvement, South China Botanical Garden, Chinese Academy of Sciences, No. 723 Xingke Road, Tianhe District, Guangzhou 510650, China; University of Chinese Academy of Sciences, No. 19A Yuquan Road, Beijing 100049, China; Guangdong Provincial Key Laboratory of Applied Botany & Key Laboratory of South China Agricultural Plant Molecular Analysis and Genetic Improvement, South China Botanical Garden, Chinese Academy of Sciences, No. 723 Xingke Road, Tianhe District, Guangzhou 510650, China; Guangdong Provincial Key Laboratory of Applied Botany & Key Laboratory of South China Agricultural Plant Molecular Analysis and Genetic Improvement, South China Botanical Garden, Chinese Academy of Sciences, No. 723 Xingke Road, Tianhe District, Guangzhou 510650, China; Guangdong Provincial Key Laboratory of Applied Botany & Key Laboratory of South China Agricultural Plant Molecular Analysis and Genetic Improvement, South China Botanical Garden, Chinese Academy of Sciences, No. 723 Xingke Road, Tianhe District, Guangzhou 510650, China; University of Chinese Academy of Sciences, No. 19A Yuquan Road, Beijing 100049, China; Guangdong Provincial Key Laboratory of Applied Botany & Key Laboratory of South China Agricultural Plant Molecular Analysis and Genetic Improvement, South China Botanical Garden, Chinese Academy of Sciences, No. 723 Xingke Road, Tianhe District, Guangzhou 510650, China; University of Chinese Academy of Sciences, No. 19A Yuquan Road, Beijing 100049, China; Guangdong Provincial Key Laboratory of Applied Botany & Key Laboratory of South China Agricultural Plant Molecular Analysis and Genetic Improvement, South China Botanical Garden, Chinese Academy of Sciences, No. 723 Xingke Road, Tianhe District, Guangzhou 510650, China; University of Chinese Academy of Sciences, No. 19A Yuquan Road, Beijing 100049, China; Center of Economic Botany, Core Botanical Gardens, Chinese Academy of Sciences, No. 723 Xingke Road, Tianhe District, Guangzhou 510650, China

## Abstract

Herbivore-induced plant volatiles (HIPVs) help the tea plant (*Camellia sinensis*) adapt to environmental stress, and they are also quality-related components of tea. However, the upstream mechanism regulating the herbivore-induced expression of volatile biosynthesis genes is unclear, especially at the level of epigenetic regulation. In this study, similar to the effects of a tea green leafhopper infestation, treatments with exogenous jasmonic acid (JA) and histone deacetylase inhibitors significantly increased the (*E*)-nerolidol content in tea and induced the expression of the associated biosynthesis gene *CsNES*. Furthermore, a key transcription factor related to JA signaling, myelocytomatosis 2 (CsMYC2), interacted with histone deacetylase 2 (CsHDA2) *in vitro* and *in vivo*. A tea green leafhopper infestation inhibited *CsHDA2* expression and decreased CsHDA2 abundance. Moreover, the tea green leafhopper infestation increased H3 and H4 acetylation levels in the promoter region of *CsNES*, which in turn upregulated the expression of *CsNES* and increased the (*E*)-nerolidol content. In this study, we revealed the effects of histone acetylations on the accumulation of HIPVs, while also confirming that CsHDA2–CsMYC2 is an important transcriptional regulatory module for the accumulation of (*E*)-nerolidol induced by tea green leafhoppers. The results of this study may be useful for characterizing plant aromatic compounds and the main upstream stress-responsive signaling molecules. Furthermore, the study findings will assist researchers clarify the epigenetic regulation influencing plant secondary metabolism in response to external stress.

## Introduction

Herbivore infestations are an important selection pressure for plants because they lead to the evolution of protective secondary metabolites. In response to insects or their eggs, some plants can synthesize and release volatile metabolites into the air [1]. These complex herbivore-induced plant volatiles (HIPVs) consist of diverse volatile organic compounds, including alkenes, alkanes, aldehydes, ketones, alcohols, ethers, esters, and carboxylic acids [2]. These stress-induced volatile metabolites help plants cope with adverse environmental stresses in a variety of ways [3], and they can also influence crop aroma quality. For example, the aroma of tea (*Camellia sinensis*), a cash crop widely cultivated in China, is essential to the sensory evaluation and commercial value of tea. Tea plants are under various stresses during the pre-harvest growth period and post-harvest processing, resulting in the induction of accumulation of volatile metabolites, thereby enhancing aroma quality [3]. Specifically, herbivore stress is one of the important aroma-inducing factors. A typical example of using herbivores to enhance the aroma of tea leaves is the noted oolong tea, Oriental Beauty Tea. Oriental Beauty Tea has a unique honey fruit fragrance, and is processed from fresh tea leaves infested by tea green leafhoppers (*Empoasca onukii*) [4]. To date, studies on HIPVs in tea plants have mostly focused on metabolite and transcript levels. For example, several studies on the effects of piercing–sucking insects (e.g. tea green leafhopper) and chewing insects (e.g. tea geometrid) on tea plants revealed that many volatile fatty acid derivatives, volatile phenylpropane/phenyl compounds, and volatile terpenes are produced and released after an infestation [5, 6]. The subsequent transcriptome-level research demonstrated that herbivore infestations activate the jasmonic acid (JA) signaling pathway and some secondary metabolite synthesis pathways in a process that may be primarily regulated by transcription factors or protein kinases [7]. Some stress-induced volatile synthase genes in tea plants have been cloned and analyzed to verify the functions of the encoded enzymes. Examples include the methyl salicylic acid carboxymethyltransferase gene *CsSAMT*, the JA carboxyl methyltransferase gene *CsJMT*, the β-ocimene synthase gene *CsOS2*, the (*S*)-linalool synthase genes *CsLIS1* and *CsLIS2*, the indole synthetase genes *CsTSA* and *CsTSB2*, the (*E*)-nerolidol synthase gene *CsNES*, and the α-farnesene synthase gene *CsAFS* [3, 8–10]. However, the mechanism underlying the herbivore-induced expression of these genes remains unclear.

Both endogenous (developmental) and exogenous environmental factors (biotic and abiotic stresses) induce the production of volatile metabolites in plants. For instance, upregulation of sesquiterpene synthesis genes in *Arabidopsis* floral organs correlates with flower development [11]. Moreover, wounding and low-temperature stress induced the expression of the indole synthesis gene *CsTSB2* in tea leaves, resulting in the accumulation of indole [12]. In addition to endogenous developmental factors and exogenous environmental factors, the plant hormone JA also has an essential role in the production of plant volatile metabolites. When tea leaves were treated with JA, the indole synthesis gene *CsTSB2* was significantly induced, and the corresponding volatile metabolite indole accumulated [12]. JA regulates downstream gene expression and metabolite responses, such as accumulation of volatile indole, mainly through the signaling pathway transcription factor MYC2 [12]. Moreover, epigenetic regulation has also been reported to be associated with volatile metabolite production in tea [13]. Under continuous wounding stress, H3K9Me2 and DNA methylation levels in the promoter region of *CsTSB2* decreased, thereby promoting *CsTSB2* expression, resulting in increased indole content [13]. Epigenetic regulatory mechanisms mainly include histone modification, nucleosome remodeling, DNA methylation, non-coding RNA and histone variants, which regulate the temporal and spatial expression of specific genes at the transcriptional level [14]. However, there are still relatively few reports describing the correlation between the accumulation of volatile metabolites in tea and epigenetic regulation. It is also unclear whether histone acetylation is related to the production of volatile metabolites in tea.

(*E*)-Nerolidol is an important compound that influences tea aromas, while also participating in plant resistance to abiotic and biotic stresses. Regarding its effect on food quality, (*E*)-nerolidol is a volatile compound with a floral fragrance; it is present in tea at a relatively high concentration, especially in oolong tea. Thus, (*E*)-nerolidol is considered to be a crucial aromatic component of high-quality oolong tea [15]. Additionally, (*E*)-nerolidol is an important biologically active compound with pharmacological properties (e.g. antioxidant, antibacterial, anti-ulcer, skin penetration enhancement, antitumor, analgesic, and anti-inflammatory properties) [16–19]. In plants, (*E*)-nerolidol and its derivative 4,8-dimethylnonyl-1,3,7-triene play a key role in responses to infestations by phytophagous insects [20]. Recent studies on tea plants revealed that (*E*)-nerolidol is a signaling substance that prompts defense of tea plants against pathogens and insects [21] and helps mediate responses to cold stress and plant–plant communication induced by cold stress [22]. However, it is unclear how herbivore infestations induce (*E*)-nerolidol accumulation.

In this study, an examination of tea leaves treated with histone deacetylase (HDAC) inhibitors indicated that HDACs may be related to the accumulation of (*E*)-nerolidol in tea plants following tea green leafhopper infestation. Correlation analyses and the identification of interacting proteins suggested that CsHDA2 and CsMYC2 may be important for the accumulation of (*E*)-nerolidol induced by tea green leafhoppers. Furthermore, we observed that the production of CsHDA2 and its binding to the *CsNES* promoter decreased after a tea green leafhopper infestation, and this enhanced the H4 acetylation of the *CsNES* promoter region. This was followed by the activation of *CsNES* and an increase in (*E*)-nerolidol content. The results of this study clarify the effect of histone acetylations on the accumulation of HIPVs and identified CsHDA2–CsMYC2 as an important transcriptional regulatory module for the accumulation of (*E*)-nerolidol induced by a tea green leafhopper infestation.

## Results

### Infestation by tea green leafhoppers induces accumulation of (*E*)-nerolidol and jasmonic acid and expression of related genes

**Figure 1:**
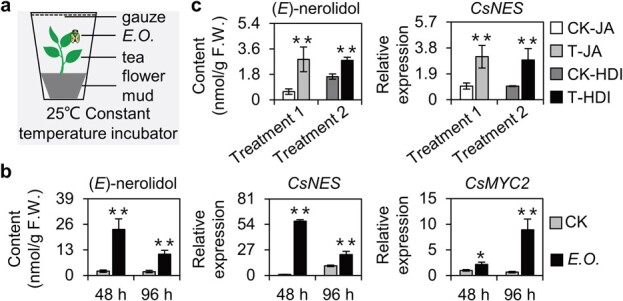
Effects of treatments of tea green leafhopper infestation, exogenous JA and HDAC inhibitors on the characteristic volatile compound (*E*)-nerolidol and corresponding gene expression levels in tea. **a** Schematic diagram of simulated indoor infestation with tea green leafhopper. *E*.*O*., *Empoasca onukii*. **b** Changes in (*E*)-nerolidol content and *CsNES* and *CsMYC2* gene expression levels after tea green leafhopper treatment. CK, control; *E*.*O*., tea green leafhopper infestation treatment. **c** Changes in (*E*)-nerolidol content and *CsNES* gene expression levels after JA (Treatment 1) and HDAC inhibitor (HDI) (Treatment 2) for 24 hours. CK-JA, solution without JA; T-JA, 2.5 mM JA; CK-HDI, solution without HDAC inhibitor; T-HDI, HDAC inhibitor. Values are expressed as mean ± standard deviation (*n* = 3). ^*^P ≤ .05, ^**^  *P* ≤ .01; control group compared with treatment group (Student’s *t*-test).

To explore the effects of a tea green leafhopper infestation on the (*E*)-nerolidol content, infestation of tea green leafhoppers on tea plants was simulated in an indoor environment (Fig. 1a). At 48 and 96 hours post-infestation, the (*E*)-nerolidol content was significantly elevated in the infested tea leaves in comparison with the control (Fig. 1b). The subsequent determination of the expression level of the (*E*)-nerolidol synthase gene, *CsNES*, indicated that it increased following infestation by tea green leafhoppers (Fig. 1b). The JA pathway is one of the most important signaling pathways for inducing HIPV production in plants. Because MYC2 is an essential transcription factor involved in the JA signaling pathway, we investigated the effect of tea green leafhoppers on *MYC2* expression in tea leaves. Infestation by tea green leafhoppers significantly induced *CsMYC2* expression (Fig. 1b). Thus, tea green leafhopper infestation promoted the accumulation of (*E*)-nerolidol and JA as well as the expression of related genes.

### Treatments with exogenous jasmonic acid and histone deacetylase inhibitors increase (*E*)-nerolidol content in tea and induce expression of *CsNES*

(*E*)-Nerolidol is a volatile secondary metabolite produced by tea plants infested with tea green leafhoppers. To explore whether its production is related to the upstream effects of JA, tea leaves were treated with JA for 24 hours and then the changes in the (*E*)-nerolidol content and in the expression of *CsNES* were analyzed. Surprisingly, the effects of the JA treatment were similar to those of the tea green leafhopper infestation. The JA treatment significantly increased the (*E*)-nerolidol content in tea leaves (Fig. 1c) while also activating the expression of *CsNES* (Fig. 1c). These findings implied that the accumulation of (*E*)-nerolidol induced by a tea green leafhopper infestation is closely related to the JA signaling pathway.

The gene expression associated with biotic stress responses is modulated by epigenetic modifications, especially histone acetylations. To verify whether histone acetylations contribute to the formation of (*E*)-nerolidol after an infestation by tea green leafhoppers, tea leaves were treated with HDAC inhibitors for 24 hours, after which the (*E*)-nerolidol content and the expression of (*E*)-nerolidol synthesis-related genes were analyzed. Consistent with the observed changes induced by the tea green leafhopper infestation, the HDAC inhibitor treatment significantly increased the (*E*)-nerolidol level in tea plants (Fig. 1c). It also induced the expression of *CsNES* (Fig. 1c), indicating that HDAC mediates the production of (*E*)-nerolidol.

### Infestation by tea green leafhoppers regulates histone deacetylase gene expression

**Figure 2:**
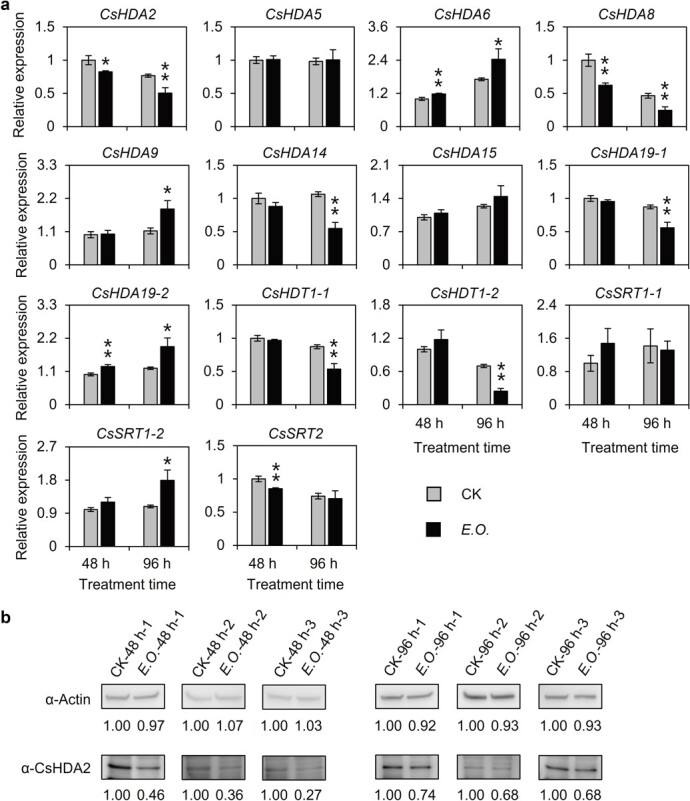
Effect of tea green leafhopper infestation on expression levels of *CsHDAC*s and CsHDA2 protein content in tea. **a** Changes in *CsHDAC* gene expression levels after tea green leafhopper treatment. CK, control; *E*.*O*., tea green leafhopper infestation treatment. Values are expressed as mean ± standard deviation (*n* = 3).^*^P ≤ .05, ^**^P ≤ .01; control group compared with tea green leafhopper infestation treatment group (Student’s *t*-test). **b** Change in CsHDA2 protein content after tea green leafhopper treatment (*n* = 3). CK, control; *E*.*O*.: tea green leafhopper infestation treatment. Actin proteins were used as loading controls.

A recent study identified 14 HDACs in tea plants [23]. To assess whether a tea green leafhopper infestation induces the accumulation of (*E*)-nerolidol by affecting HDACs, we examined the expression of 14 HDAC genes in response to tea green leafhoppers. At two post-infestation time-points, the expression levels of most of the HDAC genes were downregulated. The exceptions were *CsHDA6* and *CsSRT1-2*, which had upregulated expression levels (Fig. 2a). Because HDACs are usually transcriptional repressors, we speculated that the HDAC genes (e.g. *CsHDA2* and *CsHDA8*) with downregulated expression levels at the two analyzed time-points likely encode regulators of (*E*)-nerolidol accumulation. These results indicate that infestation by tea green leafhoppers regulates the expression of HDAC genes.

### Infestation by tea green leafhoppers inhibits CsHDA2 protein production

A previous study involving *Arabidopsis* confirmed that JA does not regulate *AtHDA8* expression [24]. Thus, we selected CsHDA2 for subsequent analyses. We prepared an anti-CsHDA2 polyclonal antibody solution and validated it with an *Escherichia coli* expression protein (Supplementary Data Fig. S1), and then used it to detect changes in the CsHDA2 level following an infestation by tea green leafhoppers. The results indicated that the abundance of CsHDA2 decreased after the tea green leafhopper infestation, which was consistent with the *CsHDA2* expression data (Fig. 2b).

### Infestation by tea green leafhoppers influences multiple metabolic pathways

**Figure 3:**
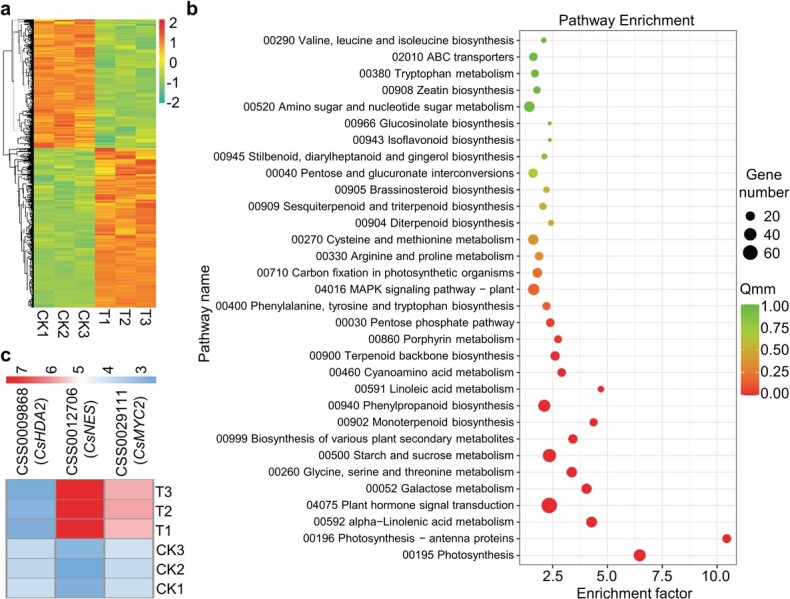
Effect of tea green leafhopper infestation on metabolic pathways in tea. **a** DEG heat map of tea leaves with and without tea green leafhopper infestation for 48 hours. Different rows represent different genes. The color represents the level of gene expression in CK (CK1, CK2, CK3) and T (T1, T2, T3). CK, control; T, tea green leafhopper infestation treatment. **b** KEGG enrichment analysis of DEGs. **c** Heat map of *CsHDA2*, *CsNES*, and *CsMYC2* in tea leaves with and without tea green leafhopper infestation for 48 hours. Different rows represent different genes. The color represents the level of gene expression in CK (CK1, CK2, CK3) and T (T1, T2, T3).

In order to evaluate more comprehensively the impact of tea green leafhopper infestation, we performed RNA-seq analysis on tea leaves after tea green leafhopper infestation for 48 hours. A total of 3034 differentially expressed genes (DEGs) were screened among all the libraries. Compared with the control group, 1353 and 1681 DEGs were downregulated and upregulated after infestation with tea green leafhoppers, respectively (Fig. 3a). Kyoto Encyclopedia of Genes and Genomes (KEGG) database enrichment analysis of the DEGs showed that plant hormone signal transduction and multiple metabolic pathways were highly enriched (Fig. 3b). The sesquiterpenoid and triterpenoid biosynthesis pathway was also included in the KEGG analysis results, which was consistent with the role of (*E*)-nerolidol as a volatile sesquiterpene (Fig. 3b). Moreover, *CsHDA2*, *CsMYC2*, and *CsNES* were all present in our DEGs, and the expression trends were consistent with previous qRT–PCR results (Fig. 3c).

### CsHDA2 is localized in the nucleus and functions as a histone deacetylase

**Figure 4:**
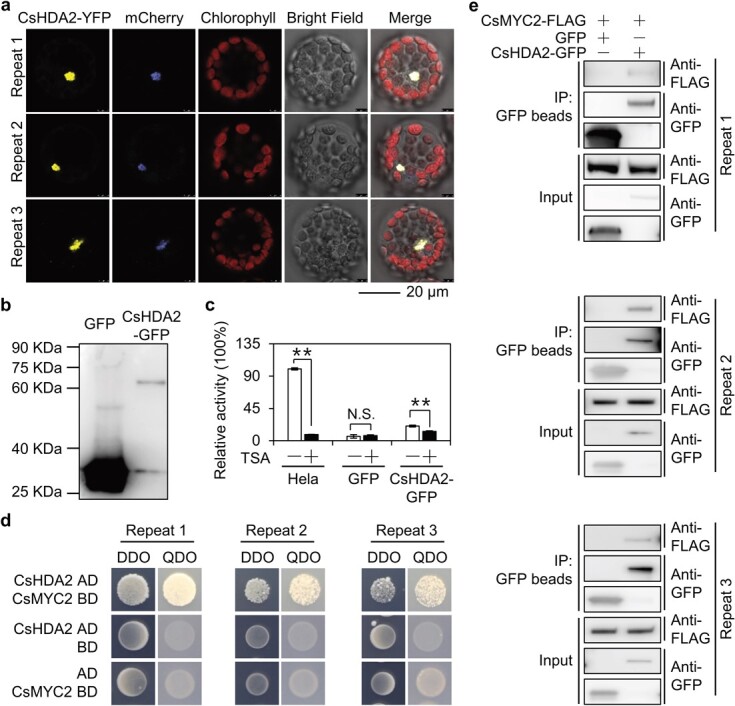
Subcellular location and function of CsHDA2 and interaction of CsHDA2 and CsMYC2 *in vivo* and *in vitro*. **a** Subcellular location of CsHDA2 in *Arabidopsis* protoplasts. **b** Affinity purification of CsHDA2–GFP expressed in tobacco leaves. CsHDA2–GFP and GFP protein were purified with anti-GFP magarose beads, and GFP antibody was used for western blot analysis. **c** Colorimetric determination of HDAC activity of purified CsHDA2–GFP. HeLa nuclear extract containing the HDAC mixture was used as a positive control. TSA was added to the sample to demonstrate the specificity of deacetylase activity. The symbols − and + represent the absence and presence of TSA, respectively. HDAC activity is expressed as the OD value measured by a spectrophotometer at 405 nm. Tobacco leaves expressing GFP were used as controls. Values are expressed as mean ± standard deviation (*n* = 3). ^**^P ≤ 0.01, N.S., no significant difference; comparison between treatment group without TSA and treatment with TSA (Student’s *t*-test). **d** A yeast two-hybrid experiment was used to analyze the interaction between CsHDA2 and CsMYC2. CsHDA2 and CsMYC2 were combined with AD and BD vectors, respectively, and co-transformed into yeast cells, and then spotted on DDO medium. Subsequently, a single colony of successfully transformed yeast was spotted on QDO medium to verify the possible interaction. DDO, SD/−Leu/−Trp; QDO, SD/−Leu/−Trp/-His/−Ade. **e** A Co-IP experiment was used to analyze the interaction between CsHDA2 and CsMYC2. Tobacco leaves co-expressing CsHDA2–GFP and CsMYC2–FLAG or GFP and CsMYC2–FLAG were immunoprecipitated with GFP antibody, and FLAG antibody was used to detect immunoblotting.

HDACs are usually transcriptional regulators in the nucleus. Accordingly, we examined the subcellular localization of CsHDA2 *in vivo*. We cloned the tea *CsHDA2* gene and fused its coding sequence to the yellow fluorescent protein (YFP) gene sequence. The resulting fusion construct was transiently expressed in *Arabidopsis* protoplasts, wherein the fluorescence of CsHDA2–YFP was detected only in the nucleus along with the nuclear localization marker mCherry signal (Fig. 4a). This suggested that CsHDA2 may be a nuclear protein that functions as a transcriptional regulator.

To further functionally characterize CsHDA2, we expressed CsHDA2–green fluorescent protein (GFP) fusion protein in tobacco leaves (Fig. 4b). HDAC activity was determined by a colorimetry-based assay. The positive control was HeLa nuclear extract containing the HDAC mixture. Additionally, the specificity of the deacetylase activity was verified by adding the HDAC inhibitor trichostatin A (TSA). Unlike the negative control, the CsHDA2–GFP protein had HDAC activities (Fig. 4c), which were significantly inhibited by trichostatin A.

### CsHDA2 interacts directly with CsMYC2 *in vivo* and *in vitro*

HDACs usually form complexes with transcription factors to perform their functions. We previously demonstrated that the accumulation of (*E*)-nerolidol induced by a tea green leafhopper infestation is related to the JA pathway and HDAC. This compelled us to determine whether CsHDA2 directly interacts with CsMYC2. We first detected the protein interaction between CsHDA2 and CsMYC2 in a yeast two-hybrid experiment. Yeast cells co-transformed with AD-CsHDA2 and BD-CsMYC2 grew on the selective medium QDO, suggesting that CsHDA2 directly interacts with CsMYC2 (Fig. 4d). Moreover, the yeast two-hybrid experiment result also showed that CsHDA8 did not interact with CsMYC2 (Supplementary Data Fig. S2).

Furthermore, CsHDA2–GFP and CsMYC2–FLAG were transiently expressed in tobacco leaves for a co-immunoprecipitation (Co-IP) analysis to verify the interaction *in vivo*. The CsMYC2–FLAG fusion protein was detected after the CsHDA2–GFP immunoprecipitation using anti-GFP antibody-coated magnetic beads (Fig. 4e). Our results indicated that CsHDA2 directly interacted with CsMYC2 both *in vivo* and *in vitro*.

### CsMYC2 promotes *CsNES* expression

**Figure 5:**
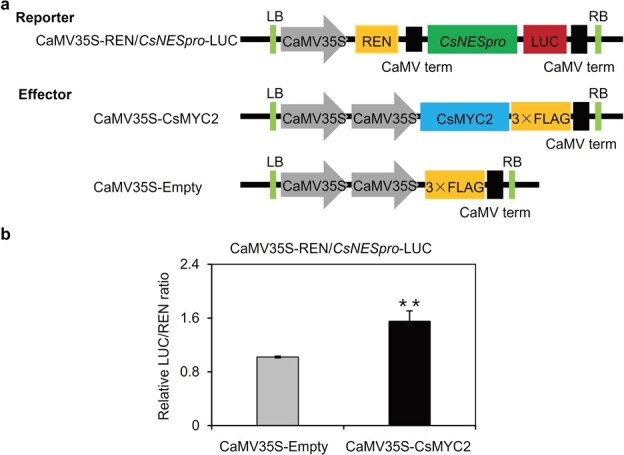
Effect of transient expression of CsMYC2 on transcriptional activity of the *CsNES* promoter. **a** Illustration of the reporter and effector plasmids used in the dual-luciferase experiment. **b** CsMYC2 had transcriptional regulatory activity on *CsNES*. Values are expressed as mean ± standard deviation (*n* = 3). ^**^P ≤ .01, Student’s *t-*test.

To evaluate whether CsMYC2 regulates *CsNES* expression, we performed a dual-luciferase reporter assay to detect transient expression. The dual-luciferase reporter plasmid contained the *CsNES* promoter fused to the LUC-encoding sequence. The REN-encoding sequence under the control of the CaMV 35S promoter was taken as an internal control, whereas the CsMYC2–FLAG plasmid was used as the effector (Fig. 5a). The expression of CsMYC2 significantly activated the *CsNES* promoter activity in comparison with the control (Fig. 5b), indicating that CsMYC2 may act as a transcriptional promoter that induces *CsNES* expression.

### Infestation by tea green leafhoppers affects binding of CsMYC2 and CsHDA2 to the *CsNES* promoter

**Figure 6:**
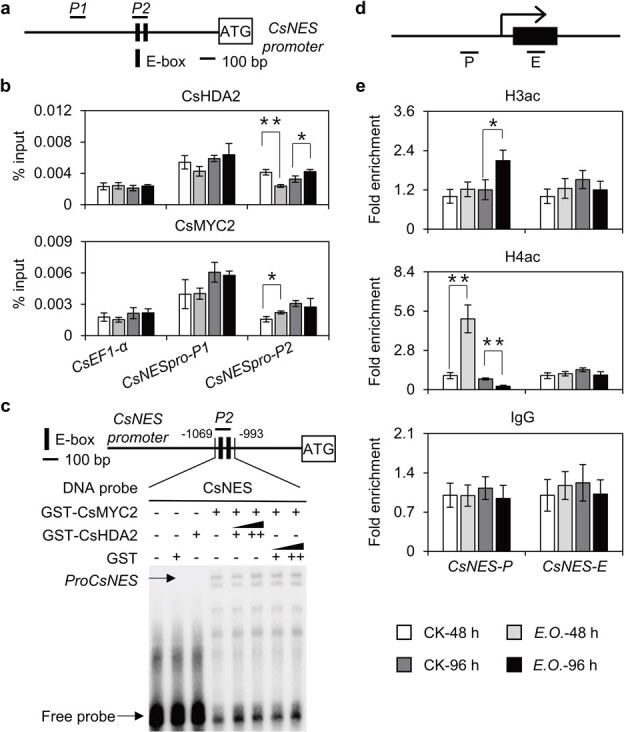
Analyses of changes in CsMYC2 and CsHDA2 binding to *CsNES* promoter and changes in H3 and H4 acetylation of *CsNES* promoter and the first exon region after tea green leafhopper infestation. **a** ChIP–qPCR detection of *CsNES* promoter site. **b** ChIP–qPCR analysis of binding changes of CsMYC2 and CsHDA2 to the *CsNES* promoter after tea green leafhopper infestation. **c** An EMSA showed that CsHDA2 did not affected the binding of CsMYC2 to the *CsNES* promoter. GST–CsMYC2 was added to the reaction. A gradient concentration of GST–CsHDA2 was applied. GST was added to the reaction as a negative control. **d** ChIP–qPCR analysis of *CsNES* promoter and the first exon region acetylation modification region. **e** ChIP–qPCR analysis of the H3 and H4 acetylation modification changes of the *CsNES* promoter (P) and the first exon (E) region after tea green leafhopper infestation. Values are expressed as mean ± standard deviation (*n* = 3). CK, control; *E*.*O*., tea green leafhopper infestation treatment. ^*^P ≤ .05, ^**^  *P* ≤ .01; control group compared with treatment group (Student’s *t*-test).

Because CsMYC2 can promote *CsNES* expression and directly interact with CsHDA2, we examined whether *CsNES* is a direct downstream target gene of CsMYC2 and CsHDA2 following a tea green leafhopper infestation. The MYC2 transcription factor usually targets the G-box (CACATG) motif or its variant (E-box) in JA-responsive gene promoters to regulate expression [25]. An analysis of the *CsNES* promoter revealed the presence of two E-box elements (Fig. 6a). Thus, we investigated the effects of an infestation by tea green leafhoppers on the binding of CsMYC2 to this region as well as to one other promoter region that lacks E-box elements. The specific binding of CsMYC2 to the P2 region (containing E-box elements) increased significantly at 48 hours post-infestation, but there were no further increases at 96 hours (Fig. 6b). We subsequently assessed the effects of a tea green leafhopper infestation on the binding of CsHDA2 to the *CsNES* promoter via a chromatin immunoprecipitation (ChIP) experiment. The results indicated that the binding of CsHDA2 to the P2 region of the *CsNES* promoter decreased significantly at 48 hours post-infestation. In contrast, the binding of CsHDA2 to the P2 regions increased at 96 hours post-infestation (Fig. 6b). These observations implied that an infestation by tea green leafhoppers may modulate *CsNES* expression by affecting the binding of CsMYC2 and CsHDA2 to the *CsNES* promoter.

Then, electrophoretic mobility shift assay (EMSA) was used to confirm the ability of CsMYC2 to bind to 3′-biotin-labeled probes, which contained two E-box motifs in the *CsNES* promoter in *vitro*. The purified GST (glutathione-*S*-transferase)–CsMYC2 fusion protein bound to the E-box, which contained a fragment of *CsNES* (Fig. 6c). Since CsHDA2 directly interacted with CsMYC2, we further determined if the interaction affected the binding of CsMYC2 to the *CsNES* promoter. Therefore, purified GST–MYC2 and GST–CsHDA2 fusion proteins were reacted with biotin-labeled probe containing the E-box motif. The result suggested that CsHDA2 did not bind to the E-box probe. Besides, the binding of CsMYC2 to the E-box probe was not changed when increasing amounts of CsHDA2 or GST were added (Fig. 6c). These findings indicated that CsHDA2 was not directly bound to the E-box region of the *CsNES* promoter, and would not inhibit the binding of CsMYC2 to the promoter of *CsNES*.

### Infestation by tea green leafhoppers increases histone H3 and H4 acetylation levels of the CsNES promoter

Previous research confirmed that CsHDA2 has HDAC activity. To explore whether the increase in *CsNES* expression caused by a tea green leafhopper infestation is related to the changes in acetylation caused by altered CsHDA2 binding, we determined the H3 and H4 acetylation levels of the *CsNES* promoter and the first exon after a tea green leafhopper infestation (Fig. 6d). Unlike the first exon, the H4 acetylation level in the *CsNES* promoter increased significantly at 48 hours post-infestation (Fig. 6e). At 96 hours post-infestation, the H3 acetylation level in the *CsNES* promoter region increased significantly, whereas the H4 acetylation level decreased significantly (Fig. 6e). Hence, the *CsNES* expression induced by the tea green leafhopper infestation was likely related to an increase in the H3 and H4 acetylation levels in the promoter region.

### Treatment with exogenous jasmonic acid changes CsMCY2 binding to the *CsNES* promoter, and treatment with histone deacetylase inhibitors increases histone acetylation levels of *CsNES*

Previous data showed that treatments with exogenous JA and HDAC inhibitors both induced the expression of *CsNES* (Fig. 1c). We then investigated how *CsNES* was regulated by upstream regulators and histone acetylation modification under exogenous JA and HDAC inhibitor treatments. After JA treatment for 24 hours, similar to tea green leafhopper infestation, the binding of CsMYC2 to the P2 region of the *CsNES* promoter was increased (Supplementary Data Fig. S3a and b). However, the binding of CsHDA2 to the *CsNES* promoter showed no significant difference after JA treatment for 24 hours (Supplementary Data Fig. S3a and b). After HDAC inhibitor treatment for 24 hours, the H3 and H4 acetylation levels of the *CsNES* promoter were increased significantly, while only the H4 acetylation level was upregulated significantly in the first exon region of *CsNES* (Supplementary Data Fig. S3c and d). Collectively, these results indicated that exogenous JA treatment regulated *CsNES* expression through CsMYC2, and HDAC inhibitor treatment regulated *CsNES* expression by affecting the H3 and H4 acetylation levels of the *CsNES* promoter and the first exon regions.

## Discussion

Herbivores are important environmental factors that cause plants to produce secondary metabolites. In this study, we revealed that changes in histone acetylation contribute to HIPV production and that CsHDA2 is a negative regulator. In the absence of tea green leafhoppers, CsHDA2 interacted with CsMYC2 and inhibited downstream *CsNES* expression through histone deacetylation. After an infestation by tea green leafhoppers, *CsHDA2* expression was downregulated. The subsequent decrease in the abundance of the encoded protein resulted in increased *CsNES* expression and the accumulation of (*E*)-nerolidol. Our findings confirmed the importance of HDACs for HIPV production.

Epigenetic modifications are closely related to the production of intrinsic secondary metabolites in diverse plants. However, most of the related research has focused on DNA methylation, histone methylation, and miRNA, with relatively few reports describing the effects of histone acetylations and specific regulators [26–34]. The current study revealed that the HDAC CsHDA2 interacts with CsMYC2, a key regulator of the JA signaling pathway (Fig. 4), to co-regulate the accumulation of the secondary metabolite (*E*)-nerolidol after tea green leafhopper infestation. In previous investigations of *Arabidopsis*, HDA6 and HDA19 were identified and thoroughly characterized as the HDACs involved in the JA pathway [24, 35–37]. In contrast, there has been less attention paid to HDA2. However, under 24-hour JA treatment the expression of *CsNES* was increased (Fig. 1c), while the binding of CsHDA2 to the *CsNES* promoter showed no significant difference (Supplementary Data Fig. S3b). These results indicated that JA-promoted *CsNES* expression might not depend on CsHDA2, or that CsHDA2 functions at an earlier time-point, which we did not detect. Earlier research suggested that HDA2 may participate in responses to cold stress [38], but its role in biotic stress responses was not elucidated. The current study indicates that *HDA2* homologs in tea plants are involved in the biotic stress response, implying that HDA2 may have diverse functions during stress responses in various species. However, whether CsHDA2 in tea plants is involved in abiotic stress responses should be investigated. Additionally, because tea plants are exposed to multiple burdens during the pre-harvest growth and post-harvest processing stages, they produce substantial amounts of secondary metabolites to cope with environmental stress [3]. There is a close relationship between epigenetic modifications and secondary metabolite production. In addition to HDACs, other potential epigenetic regulators of the accumulation of volatile metabolites induced by a tea green leafhopper infestation remain to be explored. If there are other epigenetic regulators involved, how do they interact with HDACs to co-regulate the accumulation of (*E*)-nerolidol? What is the specific mechanism underlying the coordinated or antagonistic regulation? Future research conducted to address these questions should focus on multiple networks regulating epigenetic modifications and the integrated regulatory effects of different epigenetic modifications under stress conditions.

The JA pathway is an essential signal transduction pathway for inducing HIPVs [39]. Many components of the JA signaling pathway have been identified, including a receptor (COI1), JA signaling suppressors (JAZs), and a transcription factor (MYC2) as well as its related homologs [40]. The COI1–JAZ complex functions as a co-receptor in the JA signal transduction pathway. Specifically, COI1–JAZ interacts with the JA–isoleucine conjugate, which subsequently triggers the ubiquitination and degradation of JAZs. The released MYC2 then forms homodimers or heterodimers with MYC3 or MYC4. These dimers bind to the G-box (CACATG) motif or its variants in JA-responsive gene promoters to regulate expression [25]. Previous studies on *Arabidopsis* proved that HDACs interact with multiple components of the JA signaling pathway. For example, a Co-IP analysis demonstrated that HDA6 interacts with COI1 [35]. Subsequent research indicated that HDA6 also interacts directly with JAZ1, JAZ3, and JAZ9 and inhibits EIN3/EIL1-dependent transcription [37]. In this study, we revealed that CsHDA2 interacts with CsMYC2 to co-regulate *CsNES* expression (Figs 4–6), indicating that CsHDA2–CsMYC2 is an important transcriptional regulator that controls HIPV production. Collectively, these studies suggest that, in multiple species, HDACs regulate downstream gene expression by interacting with JA signaling pathway components.

The HDACs usually form a complex with transcription factors and then inhibit transcription by regulating the acetylation level of downstream genes. However, a recent study proved that HDA9 in *Arabidopsis* deacetylates the transcription factor WRKY53 to regulate its transcriptional activity and modulate downstream gene expression [41]. Therefore, future studies should explore whether CsHDACs have diverse regulatory effects on the accumulation of HIPVs in tea. Besides, the acetylation status of histones is firmly connected to gene expression. The acetylation of histones is related to histone acetyltransferase, while the deacetylation of histones is associated with HDAC [42]. These enzymes have important roles affecting plant growth and development as well as responses to environmental stress. In *Arabidopsis*, MYC2 regulates the JA response through the formation of a complex with MED25 and the histone acetyltransferase HAC1 [43]. In the current study, the extent of the histone acetylation in the *CsNES* promoter region increased following an infestation by tea green leafhoppers (Fig. 6e). Although CsHDA2 helps regulate this process, whether histone acetyltransferases are also involved should be investigated. In addition, the binding results showed that the binding of CsHDA2 to the *CsNES* promoter decreased after 48 hours and increased after 96 hours of infestation with tea green leafhoppers (Fig. 6b), and this, combined with determinations of gene expression and (*E*)-nerolidol content (Fig. 1b), suggests that there may be a braking mechanism to prevent the overexpression of *CsNES* and the excessive accumulation of (*E*)-nerolidol by regulating the binding of CsHDA2. Due to the importance of (*E*)-nerolidol in response to herbivore infestation and the decrease in H4 acetylation level in the late stage of tea green leafhopper infestation, the elevation of H3 acetylation level was essential to maintain the expression of *CsNES*. The changes in binding of CsHDA2 to the *CsNES* promoter showed a good correspondence with the changes in H4 acetylation level, but were not consistent with the changes in H3 acetylation level (Fig. 6). These results revealed that the acetylation level may be regulated by other regulators, most likely those CsHDACs whose expression was downregulated at 96 hours (Fig. 2), indicating that different CsHDACs may be involved in the regulation of early and late responses of the same downstream gene.

*Arabidopsis* contains 18 HDACs that are divided into three families, the largest of which is the RPD3-like superfamily; HDA6, HDA19, HDA2, and HDA8 belong to this superfamily [44]. In *Arabidopsis*, JA induces the expression of *HDA6* and *HDA19* [24]. In rice, JA increases the expression of *HDA705*, which is highly homologous to *HDA6* and HDA19 [45]. Additionally, HDA6 is reportedly involved in the regulation of the JA response [36]. In our study, *CsHDA6* and *CsHDA19-2* expression levels increased significantly in tea plants infested with tea green leafhoppers (when the JA content increased significantly) (Fig. 2a). This is consistent with the results of related studies on other species. These findings imply that *HDA6* and *HDA19* expression induced by JA is common among plant species. Furthermore, JA-induced HDA6 and HDA19 functions may be conserved in multiple species, but they will need to be further explored. Interestingly, there are two *HDA19* homologs in tea. We observed that *CsHDA19-1* expression was downregulated by an increase in JA levels, whereas *CsHDA19-2* had the opposite expression trend (Fig. 2a). This differential expression of *CsHDA19-1* and *CsHDA19-2* may reflect the differences in the functions of the encoded proteins in response to JA. This possibility will need to be experimentally verified. A previous study regarding *Arabidopsis* indicated that JA does not regulate *HDA8* expression [24]. However, we observed that *CsHDA8* expression was downregulated when the JA content increased (Fig. 2a), indicating that JA-induced HDA8 functions may vary among species. Therefore, the functional characterization of the homologous genes in different species is warranted. Furthermore, although HDA6, HDA19, HDA2, and HDA8 are members of the RPD3-like superfamily, they respond differently to herbivore stress. Both HDA6 and HDA19 belong to Class I, HDA2 belongs to Class III, and HDA8 is unclassified [44]. Future studies will need to further clarify how the same upstream stress signal regulates the responses of different downstream factors as well as the interactions among different factors in the same stress response.

**Figure 7:**
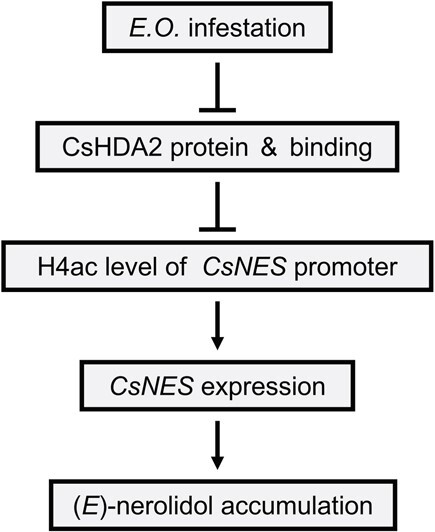
Proposed model of involvement of HDAC CsHDA2 in the formation of (*E*)-nerolidol, a characteristic volatile compound in tea, induced by infestation with tea green leafhoppers. Under tea green leafhopper infestation, the expression of *CsHDA2* was inhibited and the protein abundance was decreased. Moreover, the binding of CsHDA2 to the *CsNES* promoter was reduced, resulting in an increase in the level of H4 acetylation in the promoter region, which in turn increased the expression of *CsNES* and the content of (*E*)-nerolidol. *E*.*O*., tea green leafhopper infestation treatment. Arrows and T-bars denote positive and negative regulation, respectively.

This study explored the mechanism underlying the effect of the HDAC CsHDA2 on the production of (*E*)-nerolidol, which is a volatile compound in tea, following an infestation by tea green leafhoppers (Fig. 7). An infestation by tea green leafhoppers inhibits *CsHDA2* expression. The subsequent decreased binding of CsHDA2 to the *CsNES* promoter leads to an increase in H4 acetylation levels in the promoter region, which in turn enhances the expression of *CsNES* and the accumulation of (*E*)-nerolidol. The results of this study may be useful for clarifying the regulation of tea aromatic compound biosynthesis, and they also provide important information regarding the development of plant aromas and the associated main upstream signaling substances responsive to stress. The data presented herein may form the basis of future research on the epigenetic regulation of external stress-induced plant secondary metabolism.

## Materials and methods

### Plant materials and treatments

The tea cultivar used in this study was *C. sinensis* ‘Jinxuan’ and all the tea samples (one bud and three leaves) were collected from the Yingde tea field, China (23°N, 113°E). All treated tea samples were placed in an incubator (temperature 25°C, relative humidity 80%, light intensity 6500 lux, and 16 hours light/8 hours dark). Every replicate contained 10 tea shoots, and each treatment group had three biological repeats.

### Tea green leafhopper treatment

Tea green leafhoppers (*Empoasca onukii* Matsuda, fifth instar) were collected from the Yingde tea field, China (23°N, 113°E). The tea branches and tea green leafhoppers were collected in November 2019. Each replicate consisted of 10 tea shoots, which were cultivated in a plastic bottle, the opening of which was sealed with gauze. Then, 10 tea shoots in a bottle were treated with 40 tea green leafhoppers. Subsequently, the bottles were positioned in an incubator for 48 and 96 hours. Tea branches without infestation by tea green leafhoppers incubated in an identical situation were used as the control. Owing to the uncertainty of the infestation of tea green leafhoppers in tea, in order to achieve a better treatment effect, the treatment time for the infestation of tea green leafhoppers was relatively long.

### Jasmonic acid treatment

Tea branches (plucked in September 2020) were soaked in 2.5 mM JA solution (containing 1% ethanol for solubilization) for 24 hours. Tea branches treated with 1% ethanol–water (v/v) solution and incubated in an identical situation served as the control.

### Histone deacetylase inhibitor treatment

Tea branches (plucked in June 2018) were independently soaked in HDAC inhibitor mixed solution [containing 0.25 mM sodium butyrate, 0.25 mM SBHA (suberohydroxamic acid), 0.25 mM TSA and 0.25 mM nicotinamide; dissolved in 5% ethanol] for 24 hours. Tea branches treated with 5% ethanol–water (v/v) solution and incubated in an identical situation served as the control.

### Determination of (*E*)-nerolidol

Dichloromethane was used to extract tea samples and ethyl decanoate was used as an internal standard. After extraction, the samples were analyzed by gas chromatography–mass spectrometry analysis using a QP2010 SE (Shimadzu Corporation, Kyoto, Japan). The quantitative analysis of (*E*)-nerolidol depended on the calibration curve of the standard.

### qRT–PCR

Total RNA was extracted with a Quick Plant Total RNA Kit (Huayueyang Biotechnology Co., Ltd, Beijing, China) and reverse-transcribed into cDNA with the PrimeScript^®^ RT (TAKARA, Dalian) kit according to the manufacturer’s instructions. Roche LightCycler 480 real-time PCR instruments (Roche Applied Science, Mannheim, Germany) and SYBR Green Mix fluorescent dye reagent (Bio-Rad, USA) were used for qRT–PCR. The internal reference gene was *CsEF1-α* (GenBank no. KA280301.1). The 2^-ΔΔCt^ method was used to analyze the relative expression levels of genes [46].

### Determination of protein content

Tea samples with or without tea green leafhopper infestation were harvested for total protein extraction. The sample powder was fully homogenized with vegetable protein extraction buffer (Fude, China), then boiled and separated on Smart PAGE™ precast gels using MOPS (3-(*N*-morpholino)propanesulfonic acid) running buffer (Smart-Lifescience, China). Anti-Actin (Abbkine, USA) and anti-CsHDA2 (Huaan, China) antibodies were used as primary antibodies, and appropriate goat secondary antibodies conjugated to HRP (horseradish peroxidase) (Abbkine, USA) were used for detection. The gray-scale intensity of protein bands in western blots was analyzed by Image Lab software (Bio-Rad, USA).

### Subcellular localization

The open reading frame (ORF) of CsHDA2 was cloned into pSAT6–EYFP vector. Mesophyll protoplasts were obtained from *Arabidopsis* leaves and were enzymatically hydrolyzed in the enzyme solution at room temperature for 5 hours. The constructed vector CsHDA2–YFP (10 μg) was instantaneously transformed into *Arabidopsis* protoplasts by PEG4000 [47]. The protoplasts were observed with a confocal laser scanning microscope (Zeiss LSM 510, Carl Zeiss, Jena, Germany) after incubation at 22°C for 16 hours. The primers for subcellular vector construction are presented in Supplementary Data Table S1.

### Transient expression of protein in tobacco

The constructed vector 35S:CsHDA2–GFP was transformed into *Agrobacterium* GV3101-pSoup. The empty vector 35S:GFP was used as an internal control. A single colony was cultured in LB liquid medium (containing 50 μg/mL rifampicin and 50 μg/mL kanamycin) and shaken at 28°C overnight. Then, the *Agrobacterium* cells were collected and resuspended in suspension containing 10 mM MgCl_2_, 10 mM MES, and 150 μM acetosyringone to adjust the OD_600_ to 1.0–1.2. Then, the resuspension was stood at room temperature for 2 hours. A disposable syringe was used to inject the *Agrobacterium* liquid into the epidermis of 4-week-old tobacco leaves. After incubation for at least 48 hours, the tobacco leaves were collected and stored at −80°C.

### Histone deacetylase enzyme activity assay

The enzymatic determination method of HDAC was as described in a previous study [48]. The purified protein was collected from tobacco leaves that transiently expressed 35S:CsHDA2–GFP or 35S:GFP. Enzyme activity was determined using an HDAC enzyme activity colorimetric assay kit (K331-100, BioVision) according to the manufacturer’s instructions.

### Dual-luciferase reporter assay

For studying the transcriptional activation of CsMYC2 to *CsNES* promoter, the primers were designed with reference to the promoter sequence of *CsNES* (XM_028232453.1) based on the NCBI database (https://www.ncbi.nlm.nih.gov/). The primers for vector construction are presented in Supplementary Data Table S1. The promoter was cloned into the pGreenII 0800–LUC dual reporter vector [49], and the constructed pHB–CsMYC2–FLAG plasmid was used as an effector. The *Agrobacterium* strain GV3101-pSoup was used to transiently co-transfer the CsNES-reporter plasmid and CsMYC2-effector plasmid into tobacco leaves. After 2 days of incubation, a dual-luciferase reporter kit (Promega) and microplate reader (Tecan Infinite F50, Tecan Austria) were used to determine the activity of LUC and REN luciferases. The ratio of LUC to REN reflects the final transcription activity.

### Yeast two-hybrid experiment

The full-length ORFs of tea genes *CsHDA2*, *CsHDA8*, and *CsMYC2* were amplified separately and were cloned into pGADT7 or pGBKT7 vectors with MonClone™ Hi-Fusion Cloning Mix (catalog no. RN06004M, Monad). The paired AD and BD construct vectors were co-transformed into yeast strain AH109 by the lithium acetate method, and yeast cells were cultured on Minimal Media Double Dropouts (SD/−Leu/−Trp) at 28°C for 2 days to screen out single colonies of yeast successfully co-transformed. Then, the transformed colonies were transferred onto Minimal Media Quadruple Dropouts (SD/−Leu/−Trp/−Ade/−His) to verify whether CsHDA2/CsHDA8 and CsMYC2 interact. 3-Amino-1, 2, 4-triazole (3-AT) was added to the Minimal Media Quadruple Dropouts to repress background growth. The primers for yeast two-hybrid vector construction are presented in Supplementary Data Table S1.

### Co-immunoprecipitation

The constructed pEAQ–CsMYC2–GFP and pHB–CsHDA2–FLAG vectors were respectively transferred into *Agrobacterium* GV3101-pSoup and *Agrobacterium* co-infected 4-week-old tobacco*.* After 48 hours of culture, the tobacco leaf materials were collected and stored in liquid nitrogen. Lysis buffer (50 mM Tris–HCl at pH 7.4, 150 mM NaCl, 2 mM MgCl_2_, 1 mM DTT, 20% glycerol, 1% NP-40, 2 mM PMSF, 0.05 mM MG132) was used to extract tobacco protein. The protein supernatant obtained was added with anti-GFP magarose beads (Smart-Lifesciences, China) and incubated at 4°C for 2 hours. The beads were washed twice with washing buffer (50 mM Tris–HCl at pH 7.4, 150 mM NaCl, 2 mM MgCl_2_, 1 mM DTT, 10% glycerol, 1% NP-40, 2 mM PMSF) and were collected. The immunoprecipitated proteins were tested by western blotting with anti-GFP (Abbkine, USA) and anti-FLAG antibody (TransGen, Beijing, China).

### Chromatin immunoprecipitation–qPCR

The ChIP analysis was investigated using the reported method with little modifications [50]. The tea samples were cross-linked by 1% formaldehyde solution under vacuum for 0.5 hours. Using sonication (Scientz, 92-IID), chromatin was dissociated and cut to an average length close to 500 bp, followed by immunoprecipitation using specific antibodies, such as anti-acetyl-histone H3 (06-599, Millipore), anti-acetyl-histone H4 (06-866, Millipore), anti-CsHDA2 (Huaan, China), anti-CsMYC2 (Huaan, China), and anti-normal rabbit IgG (12–370, Millipore). The cross-linking was then reversed, and the DNA was extracted and purified with a Zymo Research Kit (D4013). The amount of each immunoprecipitated DNA fragment was determined by quantitative PCR using the gene-specific primers in Supplementary Data Table S1.

### CsHDA2 antibody validation

The ORFs of CsHDA2 were cloned into expression vector pGEX4T-3 and transferred into *E. coli* BL21 (DE3). BL21 (DE3) cells were incubated at 37°C for 1.5–2 hours to OD_600_ = 0.6. To obtain recombinant GST-tagged protein, IPTG (isopropyl β-d-thiogalactoside) was mixed in the cultures, which were incubated at 37°C for another 4 hours. Then, to obtain recombinant GST-tagged protein, the cells were harvested and resuspended in 1 × PBS buffer (2.7 mM KCl, 140 mM NaCl, 1.8 mM KH_2_PO_4_, pH 7.4, 10 mM Na_2_HPO_4_). The supernatant was collected and tested by western blot analysis with anti-GST (Abbkine, USA) and anti-CsHDA2 (Huaan, China) antibody.

### Electrophoretic mobility shift assay

The coding sequence of CsMYC2 and CsHDA2 was cloned into the pGEX4T-3 vector to yield the GST–CsMYC2 and GST–CsHDA2 fusion proteins, and the plasmids obtained were transferred into *E. coli* BL21 (DE3) cells to induce fusion proteins. In addition, empty pGEX4T-3 vectors were transformed to obtain GST tags used as control. Proteins (including GST tag, GST–CsMYC2, and GST–CsHDA2) were induced by adding IPTG and purified using glutathione beads (Smart-Lifesciences, China). Probe sequences of CsMYC2 (5′-CTACTTTCATGTGATTCTATGTTCAACTCTCCCAAGCAACATACTTACGAAAATTTTCTTGCCATTTGACAAAATG-3′ and 5′-CATTTTGTCAAATGGCAAGAAAATTTTCGTAAGTATGTTGCTTGGGAGAGTTGAACATAGAATCACATGAAAGTAG-3′) were synthesized and labeled (3′-end) with biotin. EMSA was carried out using the LightShift Chemiluminescent EMSA Kit (ThermoFisher) according to the manufacturer’s instructions.

### RNA sequencing and bioanalysis

The Quick Plant Total RNA Kit (Huayueyang Biotechnology Co., Ltd., Beijing, China) was used to extract total RNA. Sequencing libraries with 250-bp fragments were constructed using the Illumina Novseq platform with PE150 according to the standard Illumina procedure. Each sample contained three replicates.

Data analysis was provided by Bio&Data Biotechnologies Inc. (Guangzhou, China). Briefly, transcriptome raw reads were preprocessed by Fastp v.0.20.1 with default parameters for the purpose of adaptor trimming and removal of low-quality RNA-seq reads (Phred quality scores <20). Clean reads were mapped to a tea reference genome (http://tpdb.shengxin.ren/Blast.html) for further analysis. Gene expression levels were calculated using the fragments per kilobase per million reads (FPKM) method, and DEGs were defined based on the following criteria: a DESeq2-adjusted false discovery rate <0.01 and fold change >2 compared with the control samples. In addition, genes were annotated with eggNOG 5.0. The Benjamini–Hochberg test was performed to detect statistically significant enrichment of the KEGG pathway.

### Preparation of polyclonal antibody against CsMYC2 and CsHDA2

Two peptides (amino acids 288–301 and 335–348) for CsMYC2 and two peptides (amino acids 15–28 and 205–218) for CsHDA2 were chemically prepared and used to immunize rabbits to obtain antibodies (Hangzhou HuaAn Biotechnology Company, Hangzhou, China).

### Significance analysis

Student’s *t*-test was used to test whether there were any differences between the treatments. All data were expressed as mean ± standard deviation. *P* ≤ .05 and *P* ≤ 0.01 were considered as significant and extremely significant, respectively.

### Gene accession numbers

Accession numbers are as follows: *CsHDA2* (XM_028266230.1), *CsHDA5* (XM_028270527.1), *CsHDA6* (XM_028198649.1), *CsHDA8* (XM_028230298.1), *CsHDA9* (XM_028254491.1), *CsHDA14* (XM_028256101.1), *CsHDA15* (XM_028254911.1), *CsHDA19–1* (XM_028208382.1), *CsHDA19–2* (XM_028226874.1), *CsHDT1–1* (XM_028241623.1), *CsHDT1–2* (XM_028223731.1), *CsSRT1–1* (XM_028252771.1), *CsSRT1–2* (XM_028259997.1), *CsSRT2* (XM_028196821.1), *CsMYC2* (XM_028207058.1), *CsEF1-α* (KA280301.1), and *CsNES* (XM_028232453.1).

## Acknowledgements

This study was supported by financial support from the Basic Frontier Science Research Program of the Chinese Academy of Sciences (ZDBS-LY-SM032), the NSFC-Guangdong Natural Science Foundation Joint Project (U1701234), the Guangdong Basic and Applied Basic Research Foundation (2021A1515011311), the Foundation of Science and Technology Program of Guangzhou (202102020806), the Guangdong Natural Science Foundation (2016A030306039), the Regional Key Project of Science and Technology Service Network Plan of the Chinese Academy of Sciences (KFJ-STS-QYZX-093), the Hangzhou Qianjiang Special Experts Project, the Guangdong Provincial Special Fund For Modern Agriculture Industry Technology Innovation Teams (2022KJ120), and the Foundation of Key Laboratory of South China Agricultural Plant Molecular Analysis and Genetic Improvement, South China Botanical Garden, Chinese Academy of Sciences (KF202207).

## Author contributions

Z.Y. (Yang) proposed the project. D.G. contributed to protein interaction analysis, ChIP–qPCR analysis, sequence analysis, the enzyme activity assay, and the dual-luciferase reporter assay. X.Z. contributed to protein interaction analysis. S.W. contributed to sample treatments, metabolite analysis, protein analysis and subcellular localization investigation. Z.Y. (Yu) performed the analyses of metabolites and gene expression in early screening experiments. D.G. and Z.Y. (Yang) analyzed the results. D.G., Z.Y. (Yang), L.Z. and J.Q. wrote the original manuscript. Z.Y. (Yang) revised the manuscript. All authors reviewed the manuscript.

## Data availability

All sequencing data have been uploaded to the NCBI Sequence Read Archive under SRA accessions SRR19434891–SRR19434896.

## Conflict of interest

The authors have declared no conflict of interest.

## Supplementary data

Supplementary data is available at *Horticulture Research* online.

## Supplementary Material

Web_Material_uhac158
