# Supplementary material for: Involvement of histone deacetylase CsHDA2 in regulating (E)-nerolidol formation in tea (Camellia sinensis) exposed to tea green leafhopper infestation
Source: Hortic Res. 2022 Jul 28;9:uhac158. doi: 10.1093/hr/uhac158 (PMC9613726; doi:10.1093/hr/uhac158)
Supplement: Web_Material_uhac158 [file web_material_uhac158.docx]

**Title: Involvement of histone deacetylase CsHDA2 in regulating (*E*)-nerolidol formation in tea (*Camellia sinensis*) exposed to tea green leafhopper infestation**

**Authors:** Dachuan Gu ^1,^ ^†^, Shuhua Wu ^1, 2, †^, Zhenming Yu ^1,^ ^†^, Lanting Zeng ^1^, Jiajia Qian ^1, 2^, Xiaochen Zhou ^1, 2^, Ziyin Yang ^1, 2, 3, *^

***Affiliation*:**

*^1^ Guangdong Provincial Key Laboratory of Applied Botany & Key Laboratory of South China Agricultural Plant Molecular Analysis and Genetic Improvement, South China Botanical Garden, Chinese Academy of Sciences, No. 723 Xingke Road, Tianhe District, Guangzhou 510650, China*

*^2^ University of Chinese Academy of Sciences, No.19A Yuquan Road, Beijing 100049, China*

*^3^ Center of Economic Botany, Core Botanical Gardens, Chinese Academy of Sciences, No. 723 Xingke Road, Tianhe District, Guangzhou 510650, China*

^*^ Corresponding author. Ziyin Yang, Tel: +86-20-38072989, *Email address*: zyyang@scbg.ac.cn.

^†^ Co-first authors.

**
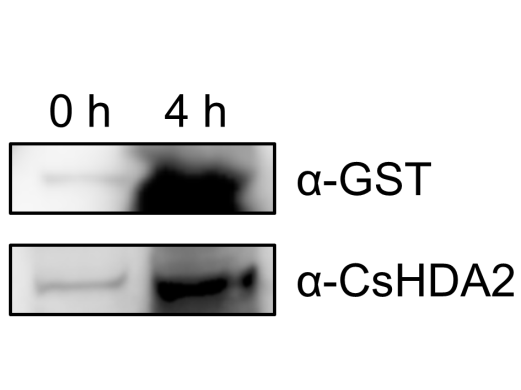
**

**Fig. S1 Western blot analysis of CsHDA2-GST expression in *E.coli* with anti-GST and anti-CsHDA2 antibody.**

The 0 h and 4 h indicated the induced time of CsHDA2-GST protein in *E.coli*.

**
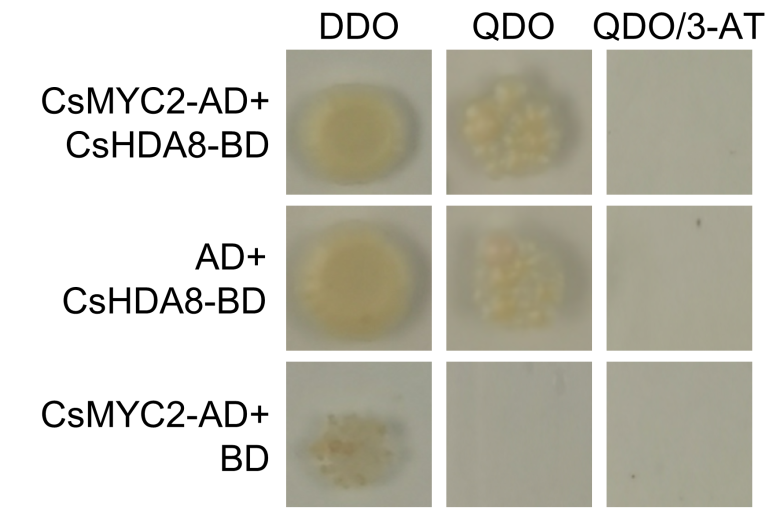
**

**Fig. S2 Analysis of the interaction between CsHDA8 and CsMYC2 using Yeast two-hybrid system.**

CsHDA8 and CsMYC2 were connected to BD and AD vectors, respectively and co-transformed into yeast cells, and then spotted on DDO medium. Subsequently, a single colony of successfully transformed yeast was spotted on the QDO medium to verify the possible interaction. DDO, SD/-Leu/-Trp; QDO, SD/-Leu/-Trp/-His/-Ade. 3-amino-1, 2, 4-triazole (3-AT) was added to the Minimal Media Quadruple Dropouts to repress the background growth.


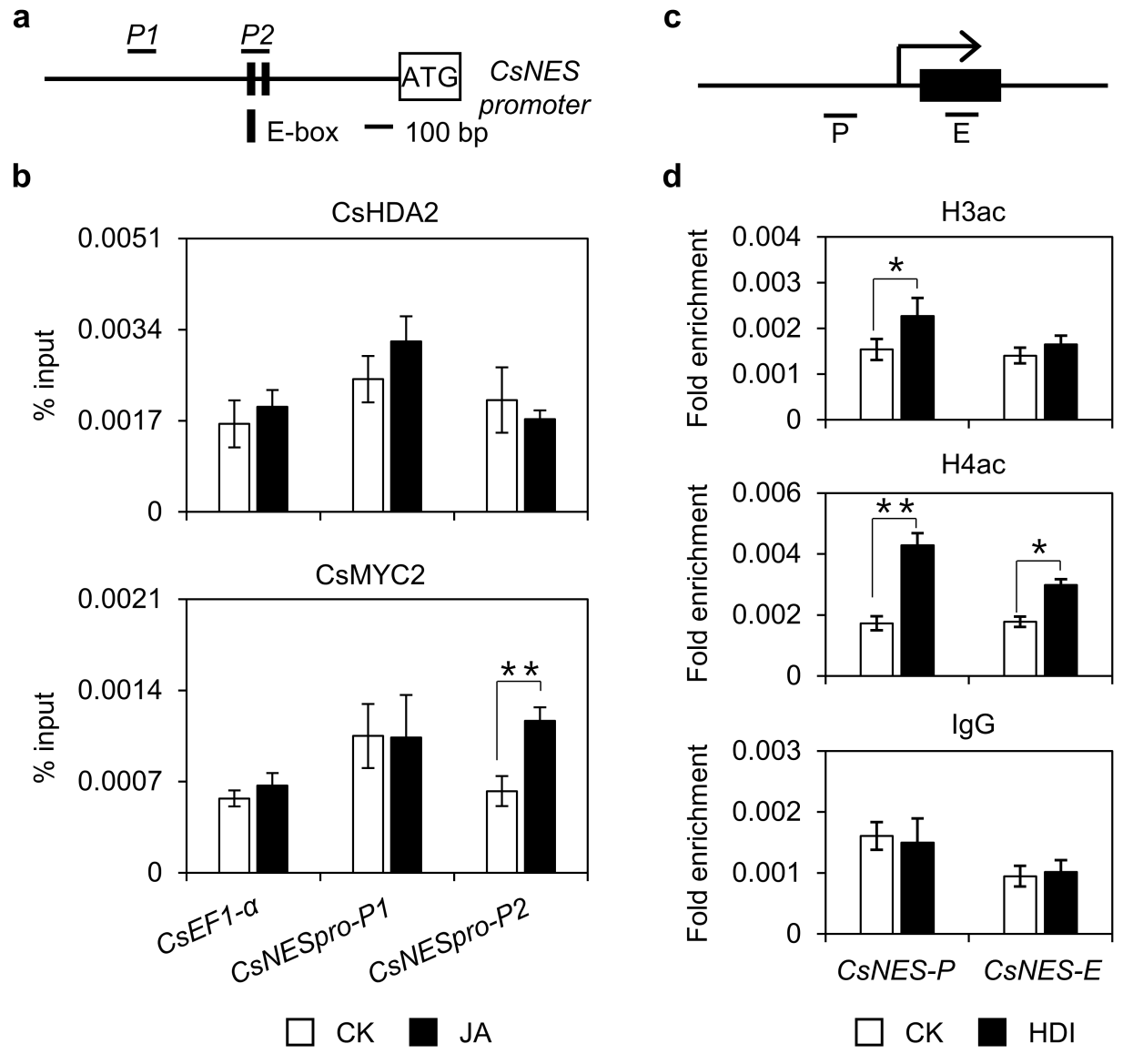


**Fig. S3 Analyses of the changes of CsMYC2 and CsHDA2 binding to *CsNES* promoter after jasmonic acid (JA) treatment and the changes of H3 and H4 acetylation levels of *CsNES* promoter and the first exon region after histone deacetylase inhibitor (HDI) treatment by ChIP-qPCR.**

(a) ChIP-qPCR detection of *CsNES* promoter site legend. (b) ChIP-qPCR analyzed the binding changes of CsMYC2 and CsHDA2 to the *CsNES* promoter after jasmonic acid (JA) treatment. (c) ChIP-qPCR analysis of *CsNES* promoter and the first exon region acetylation modification region legend. (d) ChIP-qPCR analysis of the H3 and H4 acetylation modification changes of the *CsNES* promoter (P) and the first exon (E) Region after histone deacetylase inhibitor (HDI) treatment. Values are expressed as mean ± SD (n=3). In the same time, the control group was compared with the treatment group (Student’s *t* test: **P*≤0.05; ** *P*≤0.01).

**Table S1 The primers used in the study.**

| Gene name | Accession number | Forward primer 5'-3' | Reverse primer 5'-3' |
| --- | --- | --- | --- |
| Primer pairs for qRT-PCR | | | |
| *CsEF1-α* | KA280301.1 | TTCCAAGGATGGGCAGAC | TGGGACGAAGGGGATTTT |
| *CsHDA2* | XM_028266230.1 | TGGATGGTGTTCTGGACAAA | AGCTTTGCTGCCAATATGCT |
| *CsHDA5* | XM_028270527.1 | GATTGTCCGAGGCTTATCCA | CTCAGCTCAGAAAGGGGTTG |
| *CsHDA6* | XM_028198649.1 | GATGGTGTTGAGGAGGCATT | GAACAACTGCATCTGGCTGA |
| *CsHDA8* | XM_028230298.1 | TCGAAAACCACCCTGAAAAC | CCCATTTCTATCCGCTTCAA |
| *CsHDA9* | XM_028254491.1 | TGCCATAAATGTCCCACTCA | CCGTTACCAGTAAGGGCAAA |
| *CsHDA14* | XM_028256101.1 | TGGATCAGATGCCAAAATCA | ATTATGACCCATGGCAGGAG |
| *CsHDA15* | XM_028254911.1 | ATGGAGTCATGGAGGAGTGG | AAGCAGCTTTCCACCAGAAA |
| *CsHDA19-1* | XM_028208382.1 | GATCCGGATTCTGAGATGGA | TGCAGTTGGCTCCTGTGTAG |
| *CsHDA19-2* | XM_028226874.1 | TGCTGGTGCTATGAGACTGG | TCAATATCAGGAGGCCGTTC |
| *CsHDT1-1* | XM_028241623.1 | GGGAGCGTCTACTTTTGTGG | ACAGTCACCTTTGGCAGCTT |
| *CsHDT1-2* | XM_028223731.1 | TACCACTCATCCCGAAAAGC | TTTTTCTGCACTGTGCTTGG |
| *CsSRT1-1* | XM_028252771.1 | GGAACGCCTTCATGGAATTA | TCCCCAAGCATAACACAACA |
| *CsSRT1-2* | XM_028259997.1 | AAAAAGCTTGCGCTGATGAT | AGCCAGCCTTCTCAAGTTCA |
| *CsSRT2* | XM_028196821.1 | AAAAAGCCAGCCGGATAAAT | GGGCCTTCAATTGATCTTGA |
| *CsNES* | XM_028232453.1 | CAAAGAACGTGGGCAATTTT | ACTAATACTGCGGGCACACC |
| *CsMYC2* | XM_028207058.1 | CGGCTACTACAAAGGCGAAG | GGTGTCGGTGACTTCCTCAT |
| Primer pairs for ChIP-PCR analysis | | | |
| CsEF1-α | KA280301.1 | CCATGTCGACTCTGGGAAGT | GTTCAGCCTTGAGCTTGTCC |
| CsNES-ChIP-P | XM_028232453.1 | GGGTGCTCTACTTTGGCAAC | GGATGCTGGGAAAATTCAGA |
| CsNES-ChIP-E | XM_028232453.1 | CCCACAAATCAGCAAGACCT | AGTGGGGAGTGAGACGTTTG |
| CsNESpro-ChIP-P1 | XM_028232453.1 | GGTGACCATGTGATTGATCG | ACTCCCCGGTCCAATTTTTA |
| CsNESpro-ChIP-P2 | XM_028232453.1 | GGTTGAGTGTGGGATGATGATT | CAGTCGAGCTTTCATTTTGTCA |
| Primer pairs for EMSA analysis |  |  |  |
| CsNESpro-EMSA | XM_028232453.1 | CTACTTTCATGTGATTCTATGTTCAACTCTCCCAAGCAACATACTTACGAAAATTTTCTTGCCATTTGACAAAATG | CATTTTGTCAAATGGCAAGAAAATTTTCGTAAGTATGTTGCTTGGGAGAGTTGAACATAGAATCACATGAAAGTAG |
| Primer pairs for constructions | | | |
| CsNESpro-pGreenII-0800-LUC infusion | XM_028232453.1 | GACGGTATCGATAAGCTTCTGATCCAAACGATAAC | TCTAGAACTAGTGGATCCGGATGAAGGAATTCAGG |
| CsHDA2-YFP | XM_028266230.1 | CGGTACCGCGGGCCCGGGATGTCGACCATGGCTTC | CACCATCAGGATCCCGGGAACTTTTCCTGTGGAAC |
| CsHDA2-GFP | XM_028266230.1 | CAAATTCGCGACCGGTATGTCGACCATGGCTTC | TGCTAGTCATACCGGTAACTTTTCCTGTGGAAC |
| CsHDA2-AD | XM_028266230.1 | GGAGGCCAGTGAATTCATGTCGACCATGGCTTC | CACCCGGGTGGAATTCAACTTTTCCTGTGGA |
| GST-CsHDA2 | XM_028266230.1 | GTGGATCCCCGAATTCCATGTCGACCATGGCTTC | AGTCGACCCGGGAATTCAACTTTTCCTGTGGAAC |
| CsHDA8-BD | XM_028230298.1 | CATGGAGGCCGAATTCATGTGGGGGAGTCCCAACCC | GGATCCCCGGGAATTCATTACAAAGGCGCATGTCTC |
| CsMYC2-AD | XM_028207058.1 | GGAGGCCAGTGAATTCATGACCGATTACCGGTTACC | CACCCGGGTGGAATTCCCGTGAATCGCCAATTTTGG |
| CsMYC2-BD | XM_028207058.1 | CATGGAGGCCGAATTCATGACCGATTACCGGTT | GGATCCCCGGGAATTCCCGTGAATCGCCAATTT |
| GST-CsMYC2 | XM_028207058.1 | GTGGATCCCCGAATTCCATGACCGATTACCGGTTA | AGTCGACCCGGGAATTCCCGTGAATCGCCAATTT |
| CsMYC2-FLAG | XM_028207058.1 | CTCTCTCTCAAGCTTGGATGACCGATTACCGGTTA | CCGTCACTAGTGGATCCCCGTGAATCGCCAATTT |

*EF1-α*, *encoding elongation factor 1-α*; *NES*, *(E)-nerolidol synthase*; *MYC*, *myelocytomatosis protein*; *HDA*, *histone deacetylase*; *HDT*, *HD-tuins*; *SRT*, *sirtuin*.
